# “She vaccinated my baby and that’s all…” Immunisation decision-making and experiences among refugee mothers resettled in Aotearoa New Zealand

**DOI:** 10.1186/s12889-023-16266-7

**Published:** 2023-07-13

**Authors:** Nadia A. Charania

**Affiliations:** 1 Department of Public Health, Auckland University of Technology, Auckland, New Zealand; 2 Migrant and Refugee Health Research Centre, Auckland University of Technology, Auckland, New Zealand

**Keywords:** Immunisation, Vaccine, Inequity, Child, Qualitative, Refugee, Aotearoa New Zealand

## Abstract

**Background:**

To prevent disease outbreaks, refugee children must be age-appropriately immunised. This qualitative study gained an in-depth understanding of refugee mothers’ vaccine decision-making and experiences accessing immunisation services for their children post-resettlement in Aotearoa New Zealand.

**Methods:**

An interpretive description methodology involving focus groups with refugee mothers (N = 45) was conducted in Auckland, one of the resettlement locations. Mothers were asked about their perceptions of vaccine-preventable diseases and vaccines, their experiences of attending immunisation events, and their suggestions for improvements to immunisation services. Data were analysed following the phases of reflexive thematic analysis.

**Results:**

Four themes were constructed. *Do I have a choice?* Mothers displayed pro-vaccination sentiments and parental obligation to vaccinate their children to protect their health, which underpinned their compliance with the national vaccine schedule. *Transnational vaccine perceptions and behaviours* It was evident that comparing their health experiences in their origin countries reinforced their positive perceptions of and trust in vaccines, health providers and their recommendations, the health system and government in New Zealand. Information sharing with their transnational networks had the potential to influence vaccine perceptions and behaviours in home and host countries. *Unanswered questions and concerns* Mothers discussed how many of their questions and concerns about immunisations and post-vaccine management went unanswered. *Relationships and experiences matter* Mothers stressed the importance of who vaccinated their child and how it was administered, highlighting that health providers’ demeanour and competence influence their immunisation experiences.

**Conclusions:**

Health providers are encouraged to focus on creating a positive immunisation experience for refugee background families. Qualified interpreters and provision of culturally and linguistically appropriate information are required. Transnationalism at the individual level appears to influence vaccine perceptions and behaviours among refugee-background mothers. Future research focusing on caregivers with child(ren) who are not fully vaccinated would be beneficial.

**Supplementary Information:**

The online version contains supplementary material available at 10.1186/s12889-023-16266-7.

## Introduction

Approximately 108.4 million people were forcibly displaced worldwide at the end of 2022 and children under the age of 18 years old constitute 40% of those forcibly displaced [1]. Of these forcibly displaced people, 35.3 million were refugees and 5.4 million were asylum seekers [1]. In accordance with Aotearoa New Zealand’s human rights framework, up to 1,500 refugees are resettled every year, known as quota refugees, and additional refugees are accepted under other pathways including the refugee family support category (family reunification refugees), refugee and protection status category (asylum seekers), and the new Community Organisation Refugee Sponsorship pilot category [2–4].

Disparities in vaccine-preventable disease (VPD) burden and immunisation coverage between migrants and refugees and their host populations have been reported globally [5]. To provide optimal protection against VPDs and prevent disease outbreaks, it is vital that children are age-appropriately immunised. This means that children must not only receive routine vaccinations, but also must have these vaccinations administered within the specified age period. Delayed vaccination increases the susceptibility of children to VPDs and could contribute to disease outbreaks, even if high overall coverage rates were achieved [6].

Refugee children arriving in New Zealand are noted to be particularly at risk of VPDs as many have incomplete or unknown immunisation status and few have serological immunity against certain VPDs [7]. Refugees may be under-immunised due to multiple factors, such as coming from countries with poor vaccine availability and facing interruptions of routine vaccinations while migrating [8]. Post-arrival, migrants and refugees require timely catch-up immunisations, but face many hardships as they integrate and experience disparities in the provision of preventive health care services, including immunisations [9, 10]. A complex interplay of factors can hinder access and utilisation of health and immunisation services among migrants and refugees, such as English language proficiency, cultural beliefs, limited knowledge, inadequate access to health services, and vaccine hesitancy, to name a few [11–13]. Refugees must navigate an unfamiliar health care system and establish relationships with service providers, which can be especially challenging as they simultaneously experience difficulties associated with resettling [12, 14]. Moreover, health care professionals (HCPs) have noted challenges with delivering immunisations to people with refugee backgrounds, including insufficient training related to creating catch-up immunisation schedules [15, 16].

All children under the age of 18 years, regardless of their immigration and citizenship status, are eligible to receive a series of publicly funded vaccinations as per the National Immunisation Schedule (NIS) [17]. New Zealand aims for 92% of children to be fully immunised by 24 months of age [18] and the Refugee Resettlement Strategy (RRS) aims to increase immunisation coverage amongst quota refugee children [19]. Recent retrospective cohort studies using linked health and immigration data revealed that overseas-born children with refugee backgrounds have suboptimal immunisation register enrolment and vaccination coverage rates, inferring challenges of immunisation services engaging with refugee families upon arrival and/or insufficiencies with recording overseas vaccinations [20, 21]. To date, there has been limited investigation regarding the engagement with immunisation services for children of refugee backgrounds post-resettlement in New Zealand. Against the backdrop of historically suboptimal national coverage rates and recent impact of the COVID-19 pandemic on routine childhood immunisation coverage [22, 23], it is vital to understand how caregivers of refugee backgrounds utilise and experience immunisation services for their children post-resettlement to inform refugee-specific strategies to improve and sustain high age-appropriate vaccination rates.

## Methodology and methods

The presented study is part of a multimethod study [24] comprised of a programme of quantitative and qualitative studies to explore factors associated with the access and uptake of vaccinations and develop strategies to improve age-appropriate vaccinations among refugee children post-resettlement in New Zealand. An interpretive description study [25] involving focus groups [26] was conducted to collectively explore perspectives of vaccines and vaccination experiences with caregivers of refugee backgrounds. Interpretive description is a qualitative research methodology used to understand phenomenon related to health and wellbeing to produce practical applications [25]. This methodology was appropriate for my study as the purpose was to generate meaningful knowledge from caregivers’ perspectives to inform improvements to immunisation service delivery. Ethical approval to conduct this study was granted by the Auckland University of Technology Ethics Committee (19/4).

### Researcher paradigm and positionality

I drew from an interpretive research paradigm [27] as the focus was on understanding the mothers’ perceptions and experiences of vaccinating their child(ren). A relativist ontological assumption that there are multiple constructed realities that are time and context dependent, and a constructionist epistemological position that knowledge is constructed within the (social) world we live in and thus, is subjective and value-laden, underpinned my research paradigm and influenced how I designed and undertook this research [28]. Given the intersubjective nature of the researcher-participant relationship, I was aware that facets of my social identity would impact this research, particularly how I facilitated the focus groups and analysed the data. Thus, I actively engaged in practices related to my positionality (e.g., developing a social identity map) [29] and reflexivity (e.g., reflexive researcher journal) [30]. I also reflected upon my professional knowledge and experience as a former HCP (respiratory therapist) with an educational background in health sciences, public health, and environmental sciences and over ten years of qualitative research experience conducting interviews and focus groups. When building rapport at the beginning of each focus group, I purposely shared that I was a migrant who belongs to an ethnic minority group who had recently delivered a baby in New Zealand. Being new to motherhood enabled a connection with mothers and helped to create a space for them to share their stories. Given my recent experiences, both positive and negative, with accessing immunisation services for my newborn, I was particularly attuned to the mothers’ expressions of their feelings before, during, and after vaccination appointments.

### Recruitment and data collection

Purposive sampling was used to recruit participants who were 18 years of age or over, a legal parent or guardian of a child (between 3 months and 18 years old), arrived in New Zealand on a refugee-related visa, and had lived in the country for at least six months. Caregivers were informed and invited to participate using a combination of flyers or conversations in-person or over the phone. Caregivers were recruited through the networks of local community health workers who provide various health promotion services to refugees living in Auckland, which is one of the resettlement locations. During recruitment, efforts were made to invite caregivers whose child(ren)’s vaccination status varied (i.e., fully, partially, or non-vaccinated), in addition to diversity of caregivers’ gender and cultural background. As recruitment was facilitated by various channels of the local community health workers, information about the number of caregivers approached, who declined and why was not feasible to report. The local community health workers also translated study documents and arranged the focus groups with interested caregivers. The community health workers were fluent in the languages common among the participants, including Somali, Pashto, Arabic, Burmese, and Dari, and provided consecutive language interpretation while I facilitated the focus groups in English.

The guide was based on existing literature and focused on exploring the factors that influence access and utilisation of immunisation services for their child(ren). Open-ended questions were based on: (i) the health belief model, a key theoretical framework for understanding vaccination behaviour [31], to explore their knowledge and perceptions of VPDs and vaccines, (ii) their experiences with vaccinating their child(ren) both in their countries of origin (and transit) and once resettled in New Zealand drawing from a theoretical framework about migrants’ utilisation of health services [32], and (iii) their suggestions for improving immunisation service delivery. The guide was vetted by the study’s Advisory Committee consisting of academics, health practitioners, and professionals in the field of immunisations and refugee health. Input received from nine committee members was incorporated to simplify the wording and add questions related to the role of the HCP in making vaccination decisions.

Six focus groups were conducted between July and December 2020, each ranging from four to eleven participants, and were approximately one to two hours in duration. In-person focus groups were held in convenient community venues, such as local community centres. Mothers were welcome to bring their children and various toys and activities were provided accordingly. It is important to note that data collection occurred in-between nationwide lockdowns and before the COVID-19 vaccine was available in New Zealand; thus, while the focus was not on the COVID-19 vaccine itself, perceptions of routine childhood vaccine and their experiences may have been influenced by public health measures in place, widespread (mis)information regarding the COVID-19 vaccine and media attention to vaccine hesitancy [33]. Before each discussion, participants provided informed written consent and completed a brief demographic questionnaire. While the question guide was used for all focus groups, I probed for elaboration where appropriate and was responsive to what the mothers wanted to share [28]. The focus groups were audio recorded and transcribed verbatim into electronic format by a transcribing service. Food and refreshments were provided during the focus groups, and each participant was given a voucher (koha) as a token of my appreciation for their time.

### Data analysis

As the focus of the presented research was on mothers’ vaccine perspectives and experiences, an experiential orientation to data analysis was undertaken that prioritized participants’ interpretations of their world [28]. I followed the six phases of reflexive thematic analysis [28, 34–37] using QSR NVivo® computer software (QSR International Pty Ltd., Doncaster, Victoria, Australia). I checked the transcripts for accuracy since a transcription service was used and re-read them as part of the familiarisation stage [35]. Familiarisation notes were made for each data item (i.e., focus group) and for the entire dataset, in addition to writing reflections in my researcher journal [37]. Then, while repeatedly listening to the audio recordings, I developed initial codes primarily using an inductive, fine-grain coding approach [37]. My analysis focused on both semantic and latent aspects of the data, although semantic coding predominated. Semantic (data-derived) codes provided a concise summary of caregivers’ explicit language and concepts [28]. Latent (researcher-derived) codes reflected implicit meanings and assumptions within the data which were influenced by theoretical and knowledge frameworks that I am familiar with and which guided the study (e.g., health belief model) [28]. The process of constructing prototype themes (i.e., meaningful patterns in relation to the research question) involved clustering codes [37]. Thematic maps were used to help visualise relationships between prototype themes and encompassing codes thereby checking for internal homogeneity and external heterogeneity [34]. Themes were later refined and named, and analysis iteratively continued as I drafted the findings [34].

### Trustworthiness

I used the concept of trustworthiness has an avenue to demonstrate the study’s rigour [38]. I spoke to four available community health workers who co-facilitated the focus groups to clarify my interpretations and deepen my engagement with the dataset thereby addressing credibility [38] and ensuring quality in my thematic analysis [34]. We discussed my familiarisation notes from their respective focus group and from the wider dataset, in addition to the prototype themes. This process also enabled an opportunity for me to gain more context about the findings. I also shared the preliminary findings and received input from members of the Advisory Committee. I have included a detailed description of the study context and process, including justification for philosophical, methodological, and analytical decisions made, to assist with transferability, dependability, and confirmability [38]. As reflexivity is paramount in qualitative research, I wrote in my reflexive journal to record the research progress and decisions made. I continually journaled about my reflections about how my background and values influenced judgements about coding and theming.

## Results

Forty-five mothers participated in this study with half (51%) being between 30 and 39 years old and over a third (40%) born in Afghanistan (Table 1). The majority (62%) had lived in New Zealand for a decade or more with approximately half (47%) of the mothers arriving as part of the family reunification scheme and the other half (44%) as part of the quota refugee scheme. The majority spoke languages prevalent in their respective country of origin; only eight reported that they spoke English in addition to another language. Most mothers held various educational qualifications and identified as being a housewife, student and/or professional. Collectively, the mothers cared for 173 children and all of them reported that their child(ren) were enrolled at a GP office. Most children were fully immunised for their age, with only 3 mothers reporting that their children were partially immunised, and one did not know their child’s vaccination status.

**Table 1:** Demographic characteristics of study participants (N = 45)

Demographic characteristic	Participants (%)
Age, years	
18–29 30–39 40–49 50–59 60 and over	3 (7%) 23 (51%) 10 (22%) 7 (16%) 2 (4%)
Country of Origin	
Afghanistan Iran Iraq Myanmar Saudi Arabia Somalia South Africa	18 (40%) 2 (4%) 3 (7%) 7 (16%) 2 (4%) 11 (24%) 2 (4%)
Visa Category	
Convention refugee (former asylum seeker) Family reunification refugee Quota refugee Other/Missing	1 (2%) 21 (47%) 20 (44%) 3 (7%)
Duration of stay in New Zealand, years	
1–4 years 5–9 years 10 and over	5 (11%) 12 (27%) 28 (62%)
Education	
Primary/intermediate/high school qualification Bachelor’s/Master’s/Doctorate degree No qualification Other	25 (56%) 9 (20%) 7 (16%) 4 (9%)
Household income, $NZD	
< 25,000 26,000–50,000 51,000–75,000 76,000-100,000 > 100,000 Missing	10 (22%) 16 (36%) 5 (11%) 1 (2%) 3 (7%) 10 (22%)

Four themes were constructed from the focus groups and are presented below using quotes from the participants and interpreters to illustrate examples from the data and offer additional detail.

### Do I have a choice?

Mothers were the primary vaccine decision-makers for their children and discussed how they made their decisions. During each focus group, most of the mothers shared stories about vaccine side effects, conspiracy theories, and traumatic health events perceived to be associated with vaccinations. While these stories made some mothers have “*second thoughts*” about vaccines, it did not deter them as all the mothers were supportive of receiving vaccines while they were pregnant and for their children. Vaccines were positively discussed and often referred to as “*necessary*” and “*beneficial*”.

“*So, it’s accountable, the parents, that it’s our responsibility to protect our children … if something happens to my children, I always blame myself.*” (FG#3)

Underpinning these positive vaccination views were feelings that they did not have a personal choice of whether to vaccinate their children. Vaccines were seen to protect their children from diseases, and they would worry or feel shame if they did not do everything in their power to protect their children. As such, mothers conveyed a sense of compliance with following the NIS outlined in the Well Child Health Book. Many mothers discussed how they would still vaccinate their children despite previous negative immunisation experiences, potential side effects, and not receiving enough information as the desire to protect their child’s health was of utmost importance.

*“… when she had arrived in the country … her immunisation was all up to date but because of the immunisation catch-up program, even the adults that came through [the Mangere Refugee Resettlement Centre] were just being injected with no significant information being provided. Although her belief is that it [vaccine] protects people from various diseases, it just felt like there was no ... Because they’re in New Zealand, they’ve got to get it done and that’s why people just do it without really asking questions*.” (FG#5)

Mothers also perceived certain vaccines as being mandatory in their home country, and this influenced their perception that vaccines were mandatory in New Zealand. Paradoxically, they were aware they had to give consent, but the notion of not having a choice was apparent. For example, having to show immunisation records for school enrolment reinforced the perception that they were obliged to vaccinate their children.

Mothers expressed immense trust in HCPs, the health system, and the New Zealand government more broadly. While mothers discussed vaccines with their family and friends, these conversations were primarily to share stories and experiences rather than solicit medical advice. Overwhelmingly, mothers looked to “*official*” sources for vaccine information, referring to doctors and nurses, and followed their immunisation recommendations:

“*They just ask and we accept … I’m okay with anything they [HCPs] told me. So, we trust the government, we trust everything. Whatever they say it, we are okay with that*.” (FG#3)

The trust placed in HCPs and the system appeared to be influenced by their lack of alternative options as they now live in New Zealand and cannot return to their home country.

“*We are here in New Zealand, and we have no choice but to trust them, because if you don’t trust the New Zealand system, then we don’t have access to any other medical system … So, we can’t go back to Iran. We can’t go back to Afghanistan, so we have to trust the system, and this is how we based our decision*.” (FG#6)

### Transnational vaccine perceptions and behaviours

Mothers generally perceived New Zealand to be a place where people were aware and accepting of other cultures. Amidst these perceptions of a multicultural society, there were anecdotes of times when many questioned whether they were truly accepted.

“*For example, me being in the front line all the time, being a pharmacist, the whole perception of people, they look at us and think they don’t belong here … The scarf, you know? So, they always think that. They do ask me question like, “Oh, how long you been here?“ And it kind of triggers back, “Am I accepted here? Do I have to go?“ … But in my everyday life, I feel am part of this country. I don’t remember how my life was before … I don’t have any other country. I’m from a refugee background*.” (FG#2)

Mothers’ comments reflected how their experiences in their home country influenced how they viewed and engaged with the New Zealand health system. Although they discussed some shortcomings of the health system, they were generally positive about how their health needs were addressed and they felt “*very lucky*” to be living in New Zealand.

The issue of culture and religion was discussed. Some mothers conveyed there were no cultural or religious beliefs that were against immunisations and thus this did not influence their vaccination decisions.

“*…if the vaccine is there for flu, that means that you have it, and keep the faith in your God … you shouldn’t be ignoring it [the vaccine], and you should have it*.” (FG#2)

Mothers did note that culturally there is a lot of respect for HCPs, especially doctors, in their home country and this influenced their high level of trust of HCPs in New Zealand. Comparing the competence and skills of HCPs between those in the home country and New Zealand further reinforced their trust in HCPs and their recommendations.

“*So, it’s like because of I guess the way in which our health system has developed [in home country], because it’s all privatized. And when you’re going to the doctor, you’re going for a specific reason, and the doctor’s the one that knows about diseases. And so, it’s in your best interest to follow the instructions of the doctor*.” (FG#5)

“*So, back home some people probably didn’t have the right information … but here [New Zealand], everybody’s [HCPs] professional, everybody knows what they’re doing. So, we don’t have any fear or anxiety for our children to not get the vaccine*.” (FG#6)

They noted differences in post-vaccination recommendations in New Zealand with cultural traditions practiced in their home countries. For example, several mothers spoke of cultural traditions for post-vaccine management that were passed down from previous generations, such as keeping the child warm and indoors, and not massaging the injection area or bathing them for three days.

The transnational flow of vaccine information and advice with their family and friends in their home countries also highlighted questions and concerns that arose when learning of differences of which diseases were endemic and which vaccines were included on national immunisation schedules.

“*I give my friends or my family members advice who are still back in Pakistan. I say, “Polio vaccine is not common in New Zealand, so don’t give it to your children. Don’t take your children for polio vaccine.“ … Injections are okay, but drops are not*.” (FG#6)

### Unanswered questions and concerns

There were many instances where mothers’ questions and concerns went unanswered. The most common ones related to what specific diseases vaccines protect against, vaccine side effects, and post-vaccination management. Almost all the mothers expressed some uncertainty about what specific diseases vaccines protect against. A few mothers voiced concerns about the efficacy of the annual influenza vaccine, with one mother saying she did not trust “*new*” vaccines. Moreover, the HPV vaccine was seen as a “*new concept*” as some perceived it as being only for people who are sexually active rather than protecting against cervical cancer. While some mothers were aware of immunisation milestone events and the names of some vaccines, others confused vaccines with other injections given to infants (e.g., vitamin K).

“*They don’t know the system and the language. The health professional will not go to extra step to explain it to them. They will give the jab, “Here’s a piece of paper, take it home and read it*.“ (FG#2)

“*When they say side effect, we don’t know what’s being affected*.” (FG#5).

Some mothers shared stories about arriving in New Zealand knowing their children were fully vaccinated; however, their children were given the vaccines again since they did not have appropriate vaccine documentation causing them to worry and question the safety of this practice.

“*He [her son] had all the vaccinations done there [in home country] but she didn’t bring the book. So, when they came back here, they had to do everything back again. So, it was doubled and she was a bit worried. So, the question she’s asking is, “Okay, you’ve had all the immunisations before you came to New Zealand, but because you didn’t bring or you didn’t have the certificate or anything and you come here and have it all back again, is there a problem with that? Can there be any side-effects?“*” (FG#4).

Mothers noted immunisation information inequities as all the resources were only available in English and thus, they did not receive sufficient information to support informed vaccine decision-making and post-vaccination management. A few mothers resorted to searching the internet for immunisation information, and noted the misinformation spread via social media platforms (e.g., Facebook, YouTube).

“*Everything is in English, so it’s difficult … there’s nothing that is being translated, because you are a minority group.*” (FG#4)

“*It’s scary just going to a new country and hearing “Your child has to have this [vaccine],“ and you don’t know what it is*.” (FG#2)

To overcome language barriers during immunisation appointments, mothers resorted to bringing a family member or friend who spoke English. Some mothers noted difficulties with getting the “*right*” interpreters with some questioning if they were even entitled to ask for an interpreter, especially as they were not offered an interpreter in some instances.

“*So, they [health care professional] just say, “Oh, you’re due for 15-months immunization. Could you please call the [health] center to make an appointment?“ And then, there’s nothing like, “Do you need any interpreter?“ Or “Do you need help?“ Or something. No, they don’t add that bridge. It’s just basically to the point*.” (FG#1)

“*So obviously getting an interpreter is not a problem, but getting the right interpreter is a big issue. They only probably interpret the concept or the summary of what we want to say and not interpreting the whole thing. So, this is the issue with some of the interpreters*.” (FG#6)

However, having an interpreter available for immunisation events was not seen by some mothers as an issue as they perceived that it would be difficult to access an interpreter and they were resolved to vaccinate their child.

### Relationships and experiences matter

Mothers shared various experiences and levels of satisfaction with their previous immunisation appointments for their children, noting how the services provided differed by general practice. Some mothers actively sought out practices with HCPs that were linguistically matched and this facilitated a positive immunisation experience for them.

“*The nurse at that clinic that her children had immunisation, she was multilingual so she was communicating with them in Arabic in addition of some people who want different languages. And she was showing them the meaning of vaccination, the expiry date, everything, that they are aware what they are getting for the children*.” (FG#3)

All mothers appreciated receiving reminders to book appointments (e.g., letters, email, text and/or phone) and they prioritised making it to their child’s immunisation appointment despite experiencing barriers to access, particularly related to transportation challenges. Mothers talked about how there was “*no excuse*” and would walk, take a train or bus, or find someone to drive them. They relayed that they only delayed vaccinations in instances where their child was sick, or they had a family situation to attend to.

The mothers stressed the importance of *who* vaccinates their child and *how* the vaccine is administered. The stories indicated that the immunisation experience was heavily dependent on the relationship with HCPs and their competence with vaccinating young children.

“*But with one of her children for about three months, he had like a bruise on his leg from where he’d been immunized. And that like students [nurses] that are coming in to practice, although it’s good that they’re learning, they’re probably not the best to practice on small children…*” (FG#5)

Some mothers, especially “*new Mums*” with newborns, talked about how emotional immunisation appointments were as they cried as they watched their child in pain. Their stories highlighted the important role of HCPs in supporting mothers during immunisation appointments to inform them of the process, ease any anxieties they may have, and provide appropriate advice for post-vaccine management.

Most of the mothers shared positive immunisation experiences where HCPs were welcoming and helpful. For example, a mother shared how HCPs helped as she had to bring her other three children to the vaccination appointment. However, one new mother shared a particularly negative experience of how “*a nurse comes and takes my child*” and the difficulty of watching her newborn in pain while being vaccinated. She went on to explain how she was neither informed of what to expect during the appointment nor engaged in the process.

“*She [nurse] vaccinated my baby and that’s all. And she was like, “Oh, you’re done for the day.“ I didn’t know what she was getting and, as a parent, I wanted to know. And I asked her, and she said, “Oh, it’s a process your child has to go through.“ And that’s what I got … I felt nervous. Is this the right way for my child’s health? She’s getting through this all vaccine stuff. Is it the right way? As a parent, is this a safer way for your child? So many thoughts and questions had been on my head*.” (FG#1)

Some mothers also shared negative experiences where they were stigmatised and treated unfairly by HCPs and allied staff (e.g., receptionists) during other health appointments. These experiences appeared to erode at their trust in HCPs and the health system, and importantly, altered how they accessed health care for themselves and their children in the future. One mother shared the following story,

“*Her experience is that she had cramps or something like that, so she went to the clinic and told the doctor and said she needs an interpreter and he said, “No, you don’t call an interpreter.” So, she couldn’t do anything … They weren’t friendly and things like that. So, next time … she went to the emergency and if it [was for] a child, then she’ll go to Starship [children’s] Hospital*.” (FG#4)

## Discussion

This study interviewed mothers of refugee backgrounds to understand how they utilise and experience immunisation services for their children post-resettlement. This study highlighted beliefs and attitudes that underpinned their vaccine decision-making, behaviour, and experiences to inform improvements to routine childhood immunisation service delivery.

Previous literature has highlighted barriers that impact vaccine access and acceptance among migrants and refugees [11, 13, 39–41]. Mothers in this study specifically discussed barriers relating to transportation, language proficiency, and vaccine literacy that impacted access and utilisation of immunisation services for their children. The language barrier influenced their ability to give informed consent, understand the vaccination process, and care for their children post-vaccination. The findings supported those of Shrestha-Ranjit et al. [42], which highlighted the communication difficulties experienced by Bhutanese refugee women when utilising health services and called for improved access to professional, culturally appropriate face-to-face interpreters. Moreover, as interpreters often act as unofficial health educators, education sessions can improve interpreters’ knowledge and beliefs about childhood vaccines and enable them to support parental vaccine decision-making [43]. As with previous studies, the lack of VPD and immunisation knowledge was evident [11, 13, 40, 41]. For example, throughout all but one of the focus groups, participants resorted to asking me medical questions about vaccine recommendations and side effects. Mothers specifically raised questions about the “new” HPV vaccine, which echoes previous research [13]. A scoping review has noted many strategies to reduce VPD burden among migrants and refugees; a common strategy to overcome language barriers was to provide appropriately matched educational materials [44]. There is a need for linguistically- and culturally appropriate consumer immunisation information to improve vaccine literacy among migrants and refugees; these resources should ideally be co-developed in partnership with migrant and refugee background communities [11, 45]. Further, many people use the internet as a source of health information, and Australian research shows that refugee-specific online resources on immunisations are insufficient [45].

Despite experiencing various barriers to accessing immunisation services, mothers were determined to vaccinate their children. All mothers demonstrated pro-vaccination sentiments and were the primary vaccine decision-makers, which supports results from a Canadian study with migrant mothers [39]. Other international research has noted that refugee-background caregivers are supportive of childhood immunisations and protecting their children is a strong motivator to vaccinate [40, 41]. Similar to previous research with east-African migrant and refugees in Australia, mothers in this study displayed little concern about vaccines [11]. Mothers in this study displayed high vaccine confidence despite limited awareness about diseases and vaccines and hearing various conspiracy theories and negative stories about side effects. Vaccine confidence has been described as a “nested set of beliefs and attitudes” reflecting trust in vaccines, vaccinators, and policymakers who recommend vaccine schedules [46].

Mothers’ high vaccine confidence and vaccination behaviour appeared to be influenced by transnationalism at the micro level, in this case referring to socio-cultural connections they maintained with people in their home country. Transnationalism generally refers to the social, cultural, economic, and/or political ties that migrants maintain with their place(s) of origin [47]. Previous literature has noted the influence of transnationalism on migrants’ health and health behaviours [48]. In this study, the perceptions and experiences of their home country’s HCPs and immunisation services appeared to have a long-standing influence on the high trust they placed in HCPs, nationally recommended vaccines, and the government in New Zealand. A Canadian study also revealed that migrant mothers expressed trust and gratitude when comparing the Canadian health system to that in their origin country [39]. Moreover, mothers’ cross-border relationships with family and friends facilitated information sharing about differences in their countries’ national vaccination programmes. Although these transnational social networks were not seen as their primary health information source, mothers inferred that these conversations could influence their vaccination behaviour and that of their transnational networks. Similarly, influenza vaccine attitudes and behaviours of Polish migrants living in Scotland were influenced by norms, values and developments in their home country [49]. In multicultural societies with a sizable migrant population, such as Auckland, migrants are likely to maintain socio-cultural ties with their transnational networks and these translational information flows may influence health behaviours both in their places of origin and destination. Supporting calls from a recent scoping review [50], research into the strength and direction of the relationship between transnationalism and vaccination behaviour among people with migrant backgrounds is warranted, especially as international migration continues to increase [51].

Vaccine hesitancy has been noted among migrant populations and influenced by knowledge, awareness, risk perceptions, health insurance status, health provider recommendation, and cultural factors [50]. Vaccine hesitancy is commonly defined using the World Health Organisation’s (WHO) definition and refers to the “delay in acceptance or refusal of vaccination despite availability of vaccination services” [52]. In this study, some mothers delayed vaccinations for their children while none outright refused. The primary reasons mothers shared for delaying vaccinations related to logistical barriers, time constraints due to competing personal or family commitments, and previous negative vaccination experiences with HCPs. Mothers in this study that delayed vaccinations may have been labelled as ‘vaccine hesitant’ even though the prevailing reasons for delaying did not indicate that they were either unsure or not supportive of childhood vaccinations, but rather reflected structural and organisational challenges they faced. More recently, vaccine hesitancy has been defined as “the motivational state of being conflicted about or opposed to getting vaccinated” without focus on the resulting vaccine behaviour (e.g., acceptance, delay, or refusal) [46]. Thus, it is important that we do not simply label under-immunised refugees as vaccine hesitant and centre discussions at the individual level (i.e., victim-blaming position). Instead, we must shift the rhetoric to focus on broader structural barriers to vaccine access and acceptance [53]. While research has shown that HCPs are aware of the competing priorities faced by refugee families [15], offering additional support to overcome structural access barriers to enable families to attend immunisation appointments would be beneficial.

Similar to previous research, mothers made vaccine decisions in a manner that did not reflect a complex process, instead mothers expressed a lack of personal choice and followed HCPs’ recommendations [39]. Given the high trust refugee mothers placed in vaccines, HCPs, the health system and government, efforts must be directed towards minimising any instance that may erode at this trust. These findings stress the importance of not only developing trusting patient-provider relationships, but also ensuring that the whole immunisation event is a positive experience for refugee families. It is particularly important that health care settings are welcoming, and HCPs are culturally-competent. These findings support calls for additional training for HCPs regarding culturally-competent care in the context of immunisation service delivery for refugee families [15]. Moreover, HCPs must be highly skilled with administering vaccines to young children. The mothers discussed how emotional immunisation appointments can be; thus, HCPs are encouraged to support mothers during the immunisation event and employ interventions to reduce the child’s pain and anxiety [54, 55].

The presented study generated novel knowledge about vaccine decision-making and immunisation experiences among refugee mothers. The study is not without some limitations though. Being a qualitative study, the results are not generalisable on a statistical basis, but rather the findings may be transferrable to other contexts. Despite efforts to recruit those with diverse vaccine perspectives, all the participants were supportive of vaccinations and thus these findings do not reflect perceptions and experiences of those with anti-vaccine sentiments who refuse vaccinations for their child(ren). To inform strategies to improve vaccination coverage rates, future research should focus on exploring the views among caregivers with child(ren) who are not fully vaccinated. Moreover, efforts were made to recruit male caregivers; however, all participants identified as female. Although, highlighting mothers’ voices is important as literature has noted the role of gender in relation to childhood immunisation status [56]. Lastly, this research involved cross-cultural focus groups with interpreters, which can potentially introduce inaccuracies when conveying the meaning and nuance of my questions and the participants’ answers [57]. To increase the credibility of this research, choosing suitable interpreters was paramount [58]; thus, community health workers who shared a similar migration background and were linguistically- and culturally matched to each group provided interpretation. Another benefit was that they were already familiar with medical terminology and research processes. Before the focus groups, we discussed the study’s aim and objectives, interview guide and important concepts [58]. Being a qualitative study that values subjectivity, I acknowledge the potential influence of the interpreters’ biases on how participants’ responses were interpreted [58]. As these health workers were trusted members within their communities, no additional time was required to build rapport; however, these pre-existing relationships may have positively or negatively influenced what participants shared.

## Conclusions

Mothers of refugee backgrounds resettled in Auckland, Aotearoa New Zealand, displayed high vaccine confidence. Despite experiencing barriers to accessing and utilising immunisation services, they were resolved to vaccinate their children. Findings highlighted the influence of translational networks on refugee mothers’ vaccine perceptions and behaviours both in origin and home countries. To overcome language barriers and facilitate informed vaccine decision-making, linguistically- and culturally appropriate consumer immunisation information is required. Additional information is also required about post-vaccine management. To further improve immunisation experiences, health providers are encouraged to create welcoming health environments, develop trusting relationships, attend training related to delivering culturally-competent care and administering vaccines to young children, and support mothers throughout the immunisation event, including answering their questions and concerns.

## Electronic supplementary material

Below is the link to the electronic supplementary material.

Supplementary Material 1

## Data Availability

The datasets generated and analysed during the current study are not publicly available due to privacy and ethical reasons. The collected data is of a sensitive and personal nature, and was collected from participants on the basis that strict confidentiality would be maintained. Data can be available from the corresponding author on reasonable request and will require completion of relevant confidentiality agreements.
