# Supplementary material for: “She vaccinated my baby and that’s all…” Immunisation decision-making and experiences among refugee mothers resettled in Aotearoa New Zealand
Source: BMC Public Health. 2023 Jul 13;23:1349. doi: 10.1186/s12889-023-16266-7 (PMC10347757; doi:10.1186/s12889-023-16266-7)
Supplement: Supplementary file 1 — Supplementary Material 1 [file 12889_2023_16266_MOESM1_ESM.docx]

**Supplementary file 1 – Question guide for focus groups**

*Building rapport*

1. Can you please start by telling me a bit about yourself and your family?

*Knowledge, attitudes, and practices about vaccine-preventable diseases and vaccines*

1. What do you know about diseases that can be prevented by getting vaccinations

(e.g., measles, chickenpox, whooping cough, polio, etc.)?

1. In general, what do you know about vaccines and how they work (e.g., MMR, etc.)?

*Awareness, utilisation, and experiences of immunisation services in Aotearoa New Zealand (NZ)*

1. Can you please tell me about health/vaccination services available in your

Country of Origin? How does this compare to health/vaccination services in NZ?

1. What information have you seen or been told about vaccines and where to get them

for your child(ren) in NZ?

1. *Prompts*: where did the information come from - who/which organisation was involved, what information was/not helpful, role of internet searches, role of social media, etc.

1. Please tell me about how you made the decision to vaccinate your child(ren) once in NZ.
2. What factors made you either accept, delay, or decline recommended vaccines for your child(ren)?

1. Can you please tell me about your experience(s) with vaccinating your child(ren) in NZ?
2. Prompt for information about scheduling the appointment (before), preparing and getting to the appointment (during), and post-vaccine management (after)
3. What would make future immunisation appointments better/easier for you and your child(ren)?

1. What would an ideal immunisation service look like? What would ideal educational information about vaccinations look like (format, language, etc.)?

1. Do you have anything else to add about your experience with vaccinating your children in NZ?
